# Validation of a Set of Clinical Criteria for the Diagnosis of Secondary Progressive Multiple Sclerosis

**DOI:** 10.3390/brainsci14111141

**Published:** 2024-11-14

**Authors:** Alin Ciubotaru, Daniel Alexa, Cristina Grosu, Lilia Böckels, Ioana Păvăleanu, Alexandra Maștaleru, Maria Magdalena Leon, Roxana Covali, Emanuel Matei Roman, Cătălina Elena Bistriceanu, Cristina Mihaela Ghiciuc, Doina Azoicăi, Emilian Bogdan Ignat

**Affiliations:** 1 Department of Neurology, “Grigore T. Popa” University of Medicine and Pharmacy, 700115 Iași, Romania; alinciubotaru94@yahoo.com (A.C.); daniel.alexa@umfiasi.ro (D.A.); cristina.grosu@umfiasi.ro (C.G.); catalina-elena.bistriceanu@umfiasi.ro (C.E.B.); emilian.ignat@umfiasi.ro (E.B.I.); 2 Department of Neurology, Rehabilitation Hospital, 700661 Iași, Romania; lilia.bockels@email.umfiasi.ro; 3 Mother and Child Department, “Grigore T. Popa” University of Medicine and Pharmacy, 700115 Iași, Romania; ioana-m-pavaleanu@umfiasi.ro; 4 Department of Medical Specialities I, “Grigore T. Popa” University of Medicine and Pharmacy, 700115 Iași, Romania; maria.leon@umfiasi.ro; 5 Department of Radiology, Biomedical Engineering Faculty, “Grigore T. Popa” University of Medicine and Pharmacy, 700115 Iași, Romania; ana.covali@umfiasi.ro; 6 Department of Cardiology, Sf. Spiridon Hospital, 700111 Iași, Romania; emanuel-matei.roman@email.umfiasi.ro; 7 Elytis Hospital Hope, 43A Gheorghe Saulescu Street, 700010 Iași, Romania; 8 Department of Morpho-Functional Sciences II—Pharmacology and Clinical Pharmacology, “Grigore T. Popa” University of Medicine and Pharmacy, 700115 Iași, Romania; c_ghiciuc@yahoo.com; 9 Department of Preventive Medicine and Interdisciplinarity, “Grigore T. Popa” University of Medicine and Pharmacy, 700115 Iași, Romania; doina.azoicai@umfiasi.ro

**Keywords:** multiple sclerosis, disease progression, validation criteria, expert consensus

## Abstract

**Background/Objectives**: Multiple sclerosis (MS) is a chronic inflammatory disease of the central nervous system characterized by progressive impairment of neuronal transmission due to focal demyelination. The most common form is RRMS (relapsing-remitting multiple sclerosis), which, under the influence of certain factors, can progress to SPMS (secondary progressive multiple sclerosis). Our study aimed to validate the criteria proposed by a working group of the Romanian Society of Neurology versus the criteria proposed by a group of experts from Spain, Karolinska, and Croatia concerning the progression from RRMS to SPMS. **Methods**: This was done by gathering epidemiological data (age, gender) and by applying clinical tests such as the 9HPT (9-hole peg test), 25FWT (25-foot walk test), and EDSS (expanded disability status scale) tests and the SDMT test (symbol digit modalities test). The present research is a cohort study that included a number of 120 patients diagnosed with MS according to the McDonald Diagnostic Criteria 2017. The study was carried out between January 2023 and April 2024, including patients hospitalized in the Neurology Clinic of the Clinical Rehabilitation Hospital from Iasi, Romania. The data were collected at baseline (T0) and at a 12-month interval (T1). **Results**: The statistical analysis was conducted using Kaiser–Meyer–Olkin analysis, which indicated a value of 0.683, thus validating the clinical tests used. The correlation matrix and the linear regression for all the tests showed highly significant statistical results. Furthermore, the ROC curve analysis of the criteria suggested by the working group of the Romanian Society of Neurology demonstrated that the EDSS, 9HPT, and 25FWT are highly sensitive in diagnosing SPMS, an opinion that is shared with the Spanish experts, but not with the Karolinska expert panel. Using the criteria given by the Croatian expert group in the ROC curve analysis showed that only the EDSS was strongly significant for the progression to the SPMS phase. **Conclusions**: In conclusion, all clinical methods used demonstrated that they are valid and can contribute to identifying patients with an increased risk of progression. The model proposed by the Romanian Society of Neurology working group is similar to other countries’ expert opinions and can be used to detect the risk of disease progression and establish a more tailored therapeutic management of SPMS.

## 1. Introduction

Multiple sclerosis (MS) is a chronic inflammatory disease of the central nervous system (CNS) characterized by progressive impairment of neuronal transmission due to focal demyelination and clinically manifests with a wide variety of heterogeneous symptoms [1,2,3,4,5,6,7,8,9,10]. The existence of epidemiological data on the global situation of MS is necessary to improve the identification of the main geographical and sociodemographic risk factors that may underlie the onset of pathophysiological events of the disease or influence its course.

The most common form of MS is the RR (relapsing-remitting) type, followed by the SP (secondary progressive) type; the first one can progress to the second form over time and under the influence of certain factors. Moreover, analyzing the factors involved in the progression of MS, regarding the transition from RRMS (relapsing-remitting multiple sclerosis) to SPMS (secondary progressive multiple sclerosis), needs to be evaluated more and more. In order to define the form of SPMS in a standardized way, several clinical factors have been taken into account, factors that guide the clinician in highlighting the progression of the disease, the most important being the accumulation of motor and cognitive deficits. Highlighting the relationship between these factors and the progression of MS will subsequently help to adapt diagnostic and treatment methods more sensitively and specifically.

At the moment, there are a multitude of instruments/scores that attempt to capture the progression of MS. Still, in order to define the progression to the SPMS phase in the most specific and sensitive way, it is necessary to create an algorithm or protocol that aims to use several instruments that will highlight the degree of motor and cognitive deficits [11]. Another important aspect regarding the need to define the form of SPMS is related to the high cost of investigations, and applying highly efficient tests with the lowest possible costs can positively impact the health system at the national level.

To define the degree of progression in as specific a way as possible, it is necessary to highlight the factors that will directly contribute to the worsening of the clinical picture. Several theories in the literature support the progression of MS to the SPMS form, but no nationally validated diagnostic algorithm currently defines it. Thus, in most cases, the diagnosis of SPMS is made retrospectively when a certain degree of motor and cognitive disability has already accumulated, and most times, it is irreversible [12]. Furthermore, due to an increased degree of variability of symptomatology, heterogeneity of the disease, and the duration of the disease, there are still cases of SPMS that are not accurately diagnosed. In the early stages of MS, the disease’s progression is often unnoticeable to both the physician and the patient, as it is initially marked by subtle changes in cognitive functions such as memory or attention [13]. Over time, a gradual build-up of motor deficits occurs, leading to negative impacts on both the quality of life and morbidity levels [14].

The criteria proposed by a workgroup of the Romanian Society of Neurology (not published) use changes in EDSS, 25FWT, 9HPT, and SDMT scores in order to support the diagnosis of SPMS. Changes are measured over one year retrospectively or six months prospectively and include either an EDSS increase (more than one point for an initial EDSS score below five and more than 0.5 starting from an initial EDSS above 5.5) or 20% or higher increases of completion times of either 25FWT or 9HPT.

Recent studies have explored the pathophysiological mechanisms behind MS progression, but none have definitively identified the triggers or markers specific to secondary progressive MS (SPMS) needed for targeted therapy [15]. This lack of precise markers negatively affects both physical and mental health, highlighting the need for systematic clinical monitoring to detect disease progression. While the risk of SPMS is influenced by non-modifiable factors such as age, gender, and disease duration, modifiable factors such as motor and cognitive status, treatment type, and quality of life may reflect patients’ perceptions of disease control. Regular monitoring of motor and cognitive function and specific SPMS therapies could improve patient identification and management.

### Study Hypothesis and Objectives

The study aimed to determine whether certain clinical criteria are sensitive and specific enough to identify secondary progressive MS (SPMS) in a cohort of patients at risk of progressing to this phase. The primary goal was to compare clinical data obtained using the EDSS, 9HPT, 25FWT, and SDMT tests in relation to criteria proposed by the working group of the Romanian Society of Neurology versus those from expert groups in Spain, Karolinska, and Croatia. Additionally, we sought to assess the impact of disability on motor and cognitive function by applying these tests at the start (T0) and after 12 months (T1). The secondary objective was to develop a clinical algorithm for monitoring disease progression and identifying patients at risk of SPMS. We also aimed to identify both non-modifiable factors (age, gender) and modifiable factors (motor and cognitive function, treatment type) that could contribute to the progression toward SPMS.

## 2. Materials and Methods

The present research is a cohort study that included 120 patients diagnosed with MS according to the McDonald Diagnostic Criteria 2017 [16]. The study was carried out between January 2023 and April 2024, and patients were hospitalized in the Neurology Clinic of the Clinical Rehabilitation Hospital in Iasi, Romania.

The inclusion criteria were: age more than 18 years old, a disease duration more than three years, MS diagnosis meeting McDonald criteria from 2017, only patients diagnosed with RRMS and SPMS type, EDSS score ≥ 1, and signing the informed consent. Exclusion criteria were the refusal to sign the informed consent, age less than 18 years old, “malignant” forms of MS (Balo, Marburg, tumefactive form), other types of confirmed MS such as primary progressive multiple sclerosis (PPMS), clinically isolated syndrome (CIS), radiologically isolated syndrome (RIS), a psychiatric disease that has the potential to impair judgment (schizophrenia, personality disorders, or acute psychotic disorders), no history of treatment with intravenous or oral corticosteroids in the last six months at the time of examination, active oncological disease, non-neurological motor disabling or a clinical relapse at least six months at the time of evaluation.

The present study was conducted in accordance with the approval of the Ethics Committee of the Iasi Clinical Rehabilitation Hospital and the University of Medicine and Pharmacy “G. T. Popa” Iași but also in accordance with the international regulations mentioned in the Declaration of Helsinki, 2013. No personal data were collected, all data were stored under the principle of anonymity, and the investigator undertook to use these data only for scientific purposes, being the object of study of the present work.

The data collected were age, gender, clinical assessment (9HPT, 25FWT, SDMT, EDSS), and type of treatment. For the clinical tests EDSS, 9HPT, 25 FWT, and SDMT, an analysis was performed at T0, representing the time the subject was included in the study, and T1, representing the same analysis at a 12-month interval. From a methodological point of view, the 9HPT test consists of two trials called clinical trial for each hand. For the 25FWT test, trial 1 means that the patient walks a distance of 7.20 m, and trial 2 means that the patient walks another 7.20 m.

Cognitive function status was assessed via the oral application of the SDMT test. The normative values of this test were based on the number of correctly identified symbols for 90 s. Physical disability was quantified by means of the EDSS score (an instrument that measures physical disability globally), the 9HPT (which measures upper limb motor performance), and the 25FWT (which measures lower limb motor function).

The application of the 9HPT test consists of the assessment of both the dominant and non-dominant upper limbs in two trials. The quantification of the 9HPT test consisted of measuring in seconds each trial for both the dominant and non-dominant limb. The inability to perform a trial was quantified as 0. The 25FWT test consisted of two trials of walking at a walking pace as fast as possible over a distance of 7.6 m. The test was quantified by measuring each trial in seconds, and the inability to complete the test was scored as 0.

All tests were applied according to international standardized methods, with the specification that the 9HPT was performed both in the dominant and non-dominant hand, and the inability to perform a trial was quantified as 0 s. In addition, for the SDMT, in our study, we chose the visual version with symbols because of the possibility of obtaining false negative results due to a possible motor deficit of a limb.

This study was centrally interested in the type of treatment and baseline therapy of patients with MS, both in patients who received immunomodulatory treatment (interferon beta, glatiramer acetate, teriflunomide) or immunosuppressive treatment (dimethyl fumarate, fingolimod, natalizumab, ocrelizumab, alemtuzumab, siponimod, and ladribine).

In Romania, there is no approved concensus at a national level regarding the evaluation of the progression of MS. The criteria to support the diagnosis of SPMS proposed by a workgroup of the Romanian Society of Neurology, based on literature data and experience in treating MS, involve the use of changes in EDSS, 25FWT, 9HPT, and SDMT scores in order to support the diagnosis of SPMS. Changes are measured over one year retrospectively or six months prospectively and include either an EDSS increase (more than one point for an initial EDSS score below five and more than 0.5 starting from an initial EDSS above 5.5) or 20% or higher increases of completion times of either 25FWT or 9HPT. The SDMT score was considered an additional score, and a decrease of three points between the two assessments was considered pathological.

The Spanish criteria for detection and diagnosis of disease progression in MS patients were presented at the 8th Joint ACTRIMS Conference ECTRIMS Meeting in 2020. The proposed criteria were: worsening of 2 points in the functional system, <10 years of disease, >20% time increase in T25-FW and >20% time increase in 9-HPT, and also repeated falls and worsening by at least 20% in the SDMT test [17].

The authors based their analysis on the Karolinska criteria, focusing solely on changes in EDSS scores of less than four and greater than four. Additionally, age was considered in relation to the EDSS score value as the second criterion [18].

The Croatian protocol proposes that the patients meet the Lublin criteria for SPMS, have a progressive accumulation of disability after initial relapse of remittent disease, an EDSS progression of 1 point for an EDSS score up to 5.5 and 0.5 points for an EDSS score ≥ 5.5, without relapse, a pyramidal functional system score ≥ 2, and the absence of pregnancy (Table 1) [12].

### Statistical Analysis

Data analysis was performed using SPSS (Statistical Package for the Social Sciences, Chicago, IL, USA, version 23.0). The data analyzed in this retrospective study considered the mean ± standard deviation calculation for the continuous variables with a normal distribution and the median and interquartile range for the series of variables that do not have a normal distribution. Frequency and percentage were considered for categorical variables.

For continuous variables with a normal distribution, comparisons were made using either the *t*-test for independent samples or ANOVA for comparisons of more than two separate subgroups of the data presented in the study. Nonparametric tests, specifically the Mann–Whitney test for comparing two subgroups and the Kruskal–Wallis test for comparing more than two subgroups, were used to analyze continuous variables that did not exhibit a normal distribution.

Categorical variables were compared using the Chi-square test. The relationship between various variables used in the study was assessed using the Spearman correlation test. Statistical significance was determined via a *p*-value < 0.05 for all statistical tests.

Kaiser–Meyer–Olkin (KMO) and Bartlett’s sphericity were used to analyze the validity of all the variables gathered. A KMO value above 0.5 and a significance level for the Bartlett test below 0.05 suggests a substantial correlation between the data and the collinearity of variables, indicating how strongly a single variable is correlated with others [19]. In this case, covariance was used to measure the relationship between two random variables. The metric assesses how much and to what extent the variables change together and what their influence is on two variables tested together. We also used graphs such as a scree plot to highlight whether the variables tested represent factors or principal components in an analysis. Multivariate multiple regression is a statistical method for revealing multiple possibilities between different variables tested or as a function of the dependent variables with a single set of predictor variables. The ROC curve (receiver operating characteristic) analyzes and reflects the performance of a test/diagnostic tool that is assumed to be below the chance of being sensitive and specific [20].

The application of KMO, Bartlett’s sphericity test subscale correlations, and rotated factor loadings were made to analyze the methodological validity of the variables tested in dynamics. Application of multivariate linear regression was made to analyze the model proposed by the working group of the Romanian Society of Neurology and ROC analysis of the above-mentioned criteria in relation to the criteria proposed by the group of experts from Spain, Karolinska, and Croatia.

The correlation matrix is a statistical analysis method that highlights the strength of correlation coefficients between variables in the form of a table. Each cell in the table demonstrates the correlation between two variables; in this case, it characterizes the performance of the tested variables in diagnosing SPMS. A value of the correlation matrix equal to −1 indicates a perfectly negative linear correlation between two variables, 0 indicates no linear correlation between two variables, and close to the 1 value indicates a perfectly positive linear correlation between two variables.

## 3. Results

The study group consisted of 120 patients with MS, of which 57.5% were women and 42.5% were men. The subjects included in the study were between 18 and 66 years old, with an average age of 41.37 years and ±11.36. For the 9HPT test, on the dominant hand, trial one at T0 had an average time of 31.33 s, trial two at T0 averaged 28.09 s, while at T1 (12 months apart), trial 1 averaged 29.91 s, and trial 2 averaged 30.83 s. The general characteristics regarding the studied parameters of the studied population can be observed in Table 2.

The result of Kaiser–Meyer–Olkin analysis indicates that the obtained value of 0.683 is close to 1, which shows that the validity of the factors introduced in the study (EDSS at T0 and T1, mean 9HPT with dominant and non-dominant hand at T0 and T1, mean 25FWT at T0 and T1, and SDMT at T0 and T1) are statistically adequate and also are statistically valid in terms of defining the form of SPMS. As for the analysis of Bartlett’s test of sphericity, the *p* < 0.001 value demonstrates that the above-mentioned tests statistically correlate with each other since this algorithm for detecting MS progression is adequate (Table 3).

The results of these indicators highlight a statistically significant relationship between the proposed variables, which can be used not only in detecting disease progression in the early stages but also in confirming the diagnosis of the SPMS phase.

Applying this type of analysis, we wanted to demonstrate that the variables included in the study are correlated with each other. Thus, the correlation matrix analysis confirms that these tests can be used as a clinical marker in defining MS progression (Table 4). We highlighted the most important correlations, and the results on negative correlations were SDMT T0 and at T1 in relation to EDSS at T0 and T1, mean 9HPT dominant and non-dominant hand at T0 and T1, and mean 25FWT at T0 and T1.

In the present study, the covariances have been calculated to highlight the proportion of variability in terms of the impact of each variable on the other variables introduced in the analysis. More precisely, each variable, in turn, is explained by the other factors studied. In this case, they are EDSS at T0 and T1, mean 9HPT dominant and non-dominant hand at T0 and T1, mean 25FWT at T0 and T1, and SDMT at T0 and T1 (Table 5).

The results of the initial comorbidity analysis show that each test applied (the percentage of own variability) is significant in relation to the other clinical parameters studied. The closer the value is to 1, the higher the correlation power.

A scree plot is a diagram that illustrates the tested variables on the y-axis and the corresponding number of factors influencing these variables on the x-axis. At this point, the slope of the curve indicates the number of factors influencing the analysis. The presence of the four identified factors, EDSS at T0 and T1 and 9HPT mean at T0 and T1 for the dominant and non-dominant hand, in the scree plot analysis illustrates that these factors are strongly associated with each other (Figure S1 from Supplementary Files).

**Figure 1:**
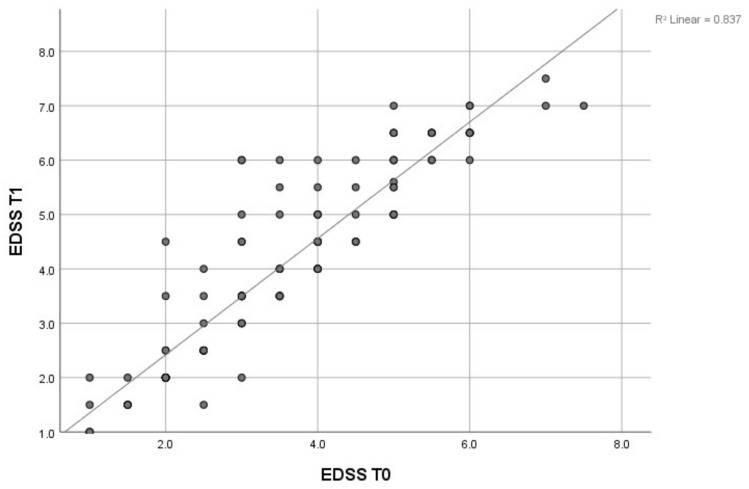
Regression line regarding the correlation between the EDSS score at T0 and T1.

The regression line’s results regarding the correlation between EDSS score at T0 and T1 showed a positive correlation, r = 0.837, which is statistically significant (Figure 1). The authors wanted to demonstrate that this proposed algorithm is valid from a methodological point of view. The application of correlation/linear regression in this case aimed to demonstrate the fact that the evaluation at T0 and T1 is specific for the inclusion of this reasoning in this algorithm. The goal was not to highlight the relationship between these variables and other factors that would contribute to the progression of the disease, but only to highlight from a statistical point of view that a one-year follow-up period is sensitive for the detection of the SPMS form.

**Figure 2:**
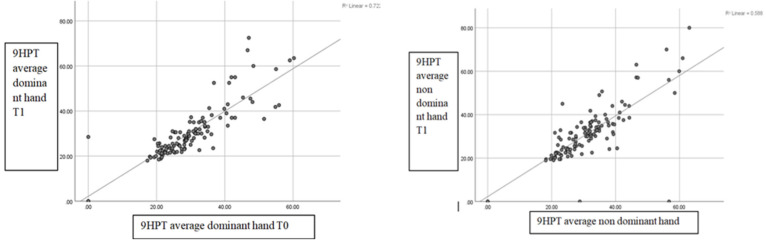

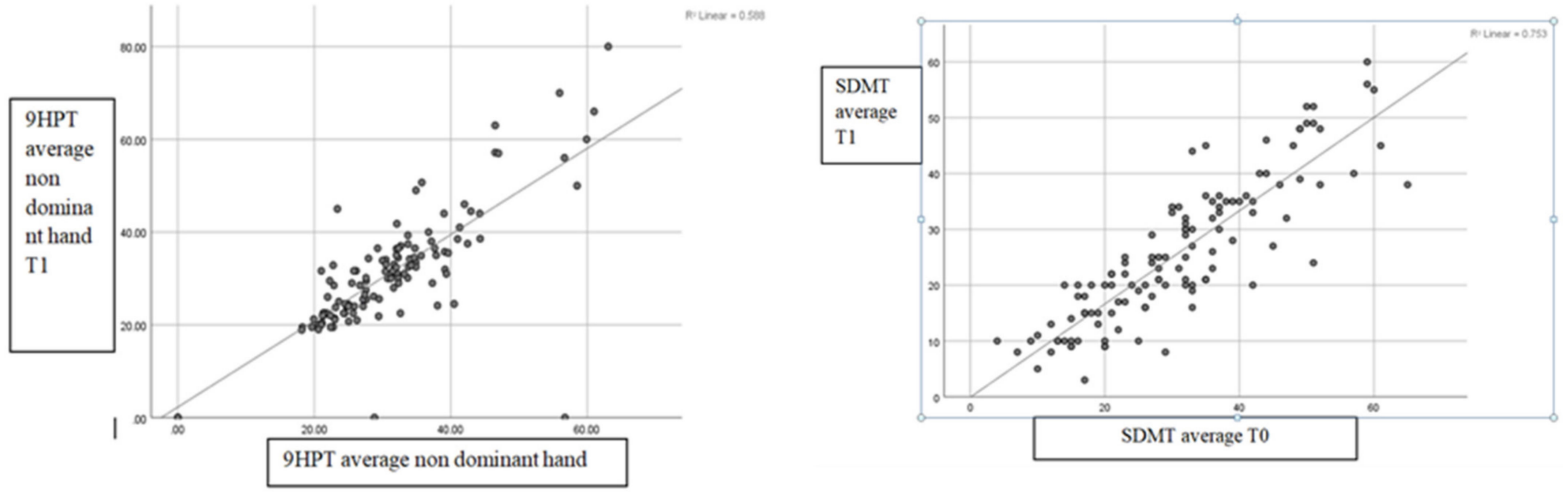
Regression line regarding the correlation at T0 and T1 for 9HPT, 25 FWT, and SDMT.

The regression analysis of the correlation between the mean 9HPT at T0 and T1 for the dominant hand and non-dominant hand showed a positive correlation of r = 0.722 and r = 0.588, respectively, which are both statistically significant. The regression line regarding the correlation between the mean 25FWT at T0 and T1 showed a positive correlation, r = 0.846, with statistical significance. The same results were obtained with the SDMT test, which showed a positive statistical significance with r = 0.753 (Figure 2).

### 3.1. Spearman Correlation Analysis in Relation to an EDSS, SDMT, 9HPT, and 25FWT Event According to the Criteria Proposed by a Working Group of the Romanian Society of Neurology

This type of correlation demonstrates that there is a direct relationship between the EDSS, SDMT, 9HPT, and 25FWT event types of the criteria proposed by a working group of the Romanian Society of Neurology, which are sensitive and specific enough to be used in the detection of the SPMS form. Statistically significant results regarding the correlation power (rho) and the level of statistical significance were obtained for all the variables proposed in this model (Table 6).

### 3.2. Multivariate Linear Regression Analysis in Relation to EDSS, SDMT, 9HPT, 25FWT, and Type of Treatment According to the Criteria Proposed by a Working Group of the Romanian Society of Neurology

In the present model, R = 0.826 (*p* < 0.001) was obtained, and the adjusted R-squared = 0.668. The Durbin–Waston test was used to analyze the degree of autocorrelation of the studied variables, which revealed a value of 1.798, the value obtained being statistically significant since a value <2 shows that the applied tests do not present a risk of autocorrelation.

The present study used multivariate linear regression analysis to reveal statistically significant data for all events except for the SDMT event. The results obtained in terms of collinearity tests were within normal limits for all variables studied, and the variance inflation factor did not exceed values greater than 10, confirming the model’s validity (Table 7).

Comparative analysis on the validation of the criteria proposed by a working group of the Romanian Society of Neurology on 9HPT, EDSS, SDMT, and 25FWT events versus the criteria proposed by the expert group from Spain, Karolinska, and Croatia to support the diagnosis of SPMS.

**Figure 3:**
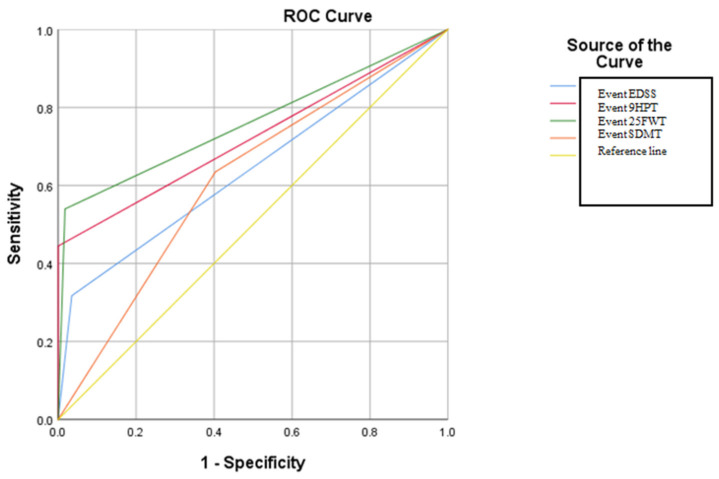
ROC curve in relation to the types of ”events” that define the form of SPMS according to the criteria of the working group of the Romanian Society of Neurology.

In order to evaluate the sensitivity and specificity of these criteria proposed by different scientific consensus groups regarding the diagnosis of SPMS, ROC curve analysis was applied. The results obtained from the ROC analysis revealed that for all types of event (9HPT, 25FWT, EDSS, and additionally SDMT), statistically significant results *p* = <0.05 were obtained. The results of the AUC analysis also revealed that the highest AUC value was recorded for the event 25FWT (AUC: 0.761), then in descending order for 9HPT (AUC: 0.722), EDSS (AUC: 0.641), and SDMT (AUC: 0.616) (see in Supplementary Files Table S1) (Figure 3). The cut-off for the ROC curve analysis, in this case, was performed according to the criteria proposed by a working group of the Romanian Society of Neurology.

**Figure 4:**
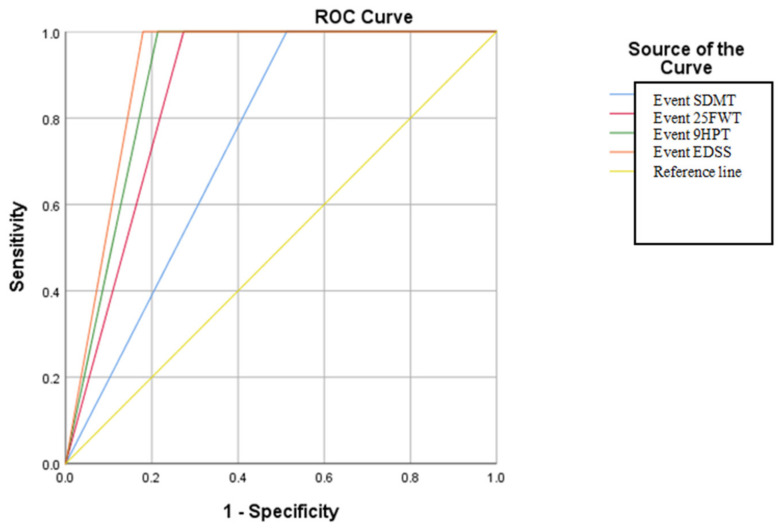
The ROC curve in relation to the types of “events” that define the type of the SPMS according to the criteria of the Spanish “expert” group.

The analysis of the criteria proposed by the Romanian Society of Neurology working group in relation to the criteria proposed by the consensus of experts in Spain has shown that the proposed model has a very high specificity and sensitivity in detecting the SPMS form. The results obtained in the present case illustrate that the analysis of the criteria ”event” proposed by the expert group of the Romanian Society of Neurology in relation to the Spanish one is valid in terms of statistical results; thus, in this case, an AUC value of 0.910 of the EDSS event was obtained, demonstrating that the global progression of the motor deficit is the most decisive factor in the diagnosis of the SPMS form. For the other types of event, AUC values were recorded in the following descending order: for event 9HPT, AUC: 0.893; 25FWT, statistically significant at *p* = <0.05; in the case of SDMT, AUC: 0.744, but without statistical significance at *p* = >0.05 (see in Supplementary Files Table S2) (Figure 4). The cut-off for the ROC curve analysis, in this case, was performed according to the criteria proposed by the group of experts from Spain.

**Figure 5:**
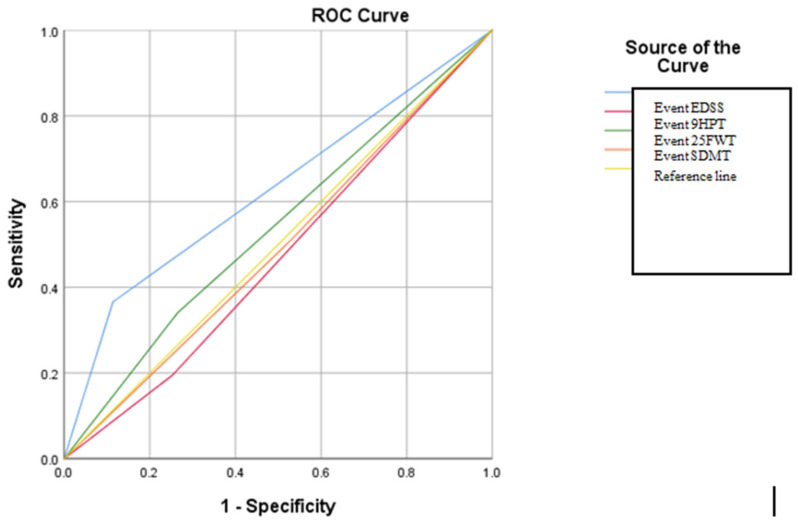
The ROC curve in relation to the types of ”event” that define the type of SPMS according to the Karolinka expert group criteria.

The analysis of the criteria proposed by the working group of the Romanian Society of Neurology in relation to the criteria proposed by the Karolinska expert consensus on the diagnosis of the SPMS phase revealed that the classification and diagnostic algorithm of the Karolinska expert consensus does not match the model proposed by the consensus of the Romanian Society of Neurology. In this case, no statistically significant data were revealed for the variables (illustrated in Table S3 from Supplementary Files), as they were statistically insignificant (Figure 5). The cut-off for the ROC curve analysis, in this case, was performed according to the criteria proposed by the Karolinksa expert group.

**Figure 6:**
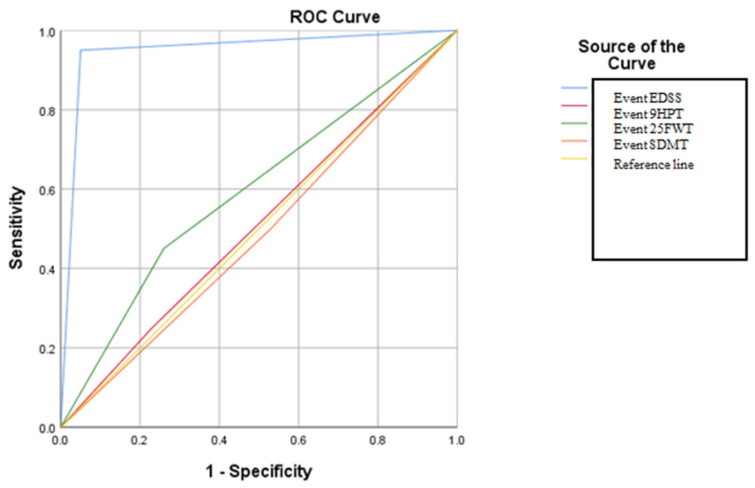
The ROC curve in relation to the types of “events” that define the shape of the SPMS according to the criteria of the Croatian expert group.

The research aims to compare the diagnostic accuracy of SPMS between the criteria established by the Romanian Society of Neurology working group and the consensus of experts from Croatia. The analysis will determine if there are any parallels in the diagnostic accuracy between these two groups of experts. The results obtained in this case showed a strong relationship with respect to the EDSS event, being the only variable in the analysis that showed data with statistical significance at *p* = <0.05 and AUC: 0.950 (Figure 6). This value demonstrates that the application of the EDSS score in the diagnostic algorithm of SPMS shows high specificity and sensitivity. No statistically significant data were evident for the other variables studied in Table S4 from Supplementary Materials. In this case, the ROC curve analysis cut-off was carried out according to the criteria proposed by the Croatian expert group.

## 4. Discussion

The primary objective of this study was to comprehensively assess the transition of MS SPMS by analyzing changes in various clinical tests, including EDSS, 9HPT, 25FWT, and SDMT. The goal was to identify a precise algorithm based on clinical markers that would effectively target the progression of the disease with high sensitivity and specificity. The global data regarding the conversion of MS to the SPMS form indicate that this occurrence is somewhat varied in the evolution of the disease. Literature data suggest that the estimated prevalence of MS is 22.42 (99% CI: 18.30, 26.95) per 100,000 patients. According to Vasanthaprasad and colleagues, there is a larger incidence of conversion to the SPMS form in Northern European countries, particularly Sweden, compared to Brazil [21].

The risks associated with the conversion of MS from the RRMS phase to the SPMS form refer to the following aspects: cognitive-attention deficit, speed of information processing, verbal fluency, visual-spatial construction, distributive attention, abstraction, and computational ability [22]. The motor impairment consists of the worsening of pre-existing motor deficits, but without a sudden worsening as is encountered in relapse, the motor function impairment, in this case, being subclinical, with a negative impact on quality of life. To evaluate these changes, in our study, a set of tests (EDSS, 9HPT, 25FWT, and SDMT) were applied to 120 patients with MS at a baseline and within a year.

At first, we aimed to check if our study hypothesis would prove valid. The Kaiser–Meyer–Olkin (KMO) is a statistical indicator that compares the “global” correlation power with the partial correlation coefficient between the studied variables. Values below 0.50 suggest that the data is inadequate for factor analysis. However, this investigation achieved a value of 0.683, indicating that the study hypothesis is statistically valid.

Another statistical analysis used to verify the consistency of the tests applied was Bartlett’s test of sphericity. It was applied to evaluate the relationship between EDSS at T0 and T1, mean 9HPT dominant and non-dominant hand at T0 and T1, mean 25FWT at T0 and T1, and SDMT at T0 and T1. The objective was to confirm the hypothesis that these clinical tests can be used to determine if there are equal variances between them, and the results indicate that the tests are uniform and have equal variances. This suggests that it may be possible to develop a clinical strategy to determine the form of SPMS.

The correlation matrix analysis results focused on applying parametric tests to measure the extent of motor and cognitive impairment in MS patients. During the investigation, a set of correlations were discovered, with each variable showing a substantial association with the other variables examined.

Our study found positive correlations between the EDSS at T0 and the mean 9HPT scores for both the dominant and non-dominant hand at T0 and T1 and the mean 25FWT scores at T0 and T1, based on the correlation matrix analysis. This demonstrates that the model being discussed is comparable to the model mentioned earlier, indicating a strong ability to accurately measure the progression of neurodegeneration, which is the main pathological process in SPMS. Negative correlations were observed between the SDMT scores at T0 and T1 and the EDSS scores at T0 and T1. This finding demonstrates that the decline in cognitive function is not directly linked to the decline in motor performance.

The correlation between the EDSS test at T0 and T1, as indicated by the linear regression result, yielded a value of r = 0.837. This value signifies that the application of this test is highly sensitive in detecting early motor deficits, making it a valuable tool for identifying the transition from SM to the SPMS phase. The EDSS score can be influenced by various conditions, such as higher body mass or vitamin D levels [15]. Although the EDSS lacks in sensitivity and specificity in measuring motor disability, it serves as a comprehensive disability score that objectively assesses the disease’s impact on the patient.

Maffezzini and colleagues found that the clinical profile of MS affects cognitive and motor functions in relation to disease duration. Results indicated that a strong cognitive reserve does not increase the risk of disease progression, particularly for cognitive decline, but a longer disease duration negatively impacts motor function. The study also showed a positive correlation between EDSS scores and measures of motor and cognitive dysfunction. Unlike Maffezzini’s findings of negative correlations in cognitive assessments, our study observed positive correlations [23].

Lorscheider and colleagues examined various clinical criteria to provide a practical definition for the form of SPMS that would be most suitable for physicians. The findings of this study indicate that having an EDSS score of 4 or higher and a pyramidal score of 2 or higher is linked to an 87% sensitivity in identifying disease progression [24]. According to many other researchers, the EDSS score appears to be a ‘universal’ test in assessing patients showing signs of progression. The modification of gait (frequency, acceleration speed, etc.) at various disease stages tends to worsen the EDSS and shows clinical progression.

In our study, the analysis of the results regarding the measurement of gait performance (25FWT at T0 and T1) showed commonality values of 0.858 and 0.935, respectively. The assessment of motor performance in the lower limbs using the 25FWT test at T0 and T1 showed that the regression line analysis of the correlation power was r = 0.846. This result strongly indicates the effectiveness of this scale in monitoring the motor function of the lower limbs.

Flachenecker and co-workers succeeded in validating the usefulness of an electronic sensor-based system aimed at monitoring motor dysfunction in patients with MS in the early stages of the disease, by using the 25FWT test and the EDSS score. The differences in mean step length, walking speed, and walking angle, including rest time and swing time, were analyzed and at the end the study revealed that lower limb motor performance is directly related to the dynamics of the EDSS score [25]. Spearman’s correlation analysis, based on the criteria proposed by the Romanian Society of Neurology working group, showed that the changes in the 25FWT had the highest correlation (rho = 0.574). This indicates that the motor deficit in the lower limbs is a crucial factor in determining the phase of SPMS. Significant values have been identified for the 9HPT, EDSS, and SDMT changes. Based on Spearman correlation research, these suggested criteria are correlated with the identification of disease progression, making them a valuable tool in clinical practice.

Creating a clinical biomarker could be valuable for tracking motor function, mainly when used alongside other clinical biomarkers to confirm the diagnosis of SPMS [26].

As for the motor function of the upper limb, our study used the 9HPT test in dynamics. The regression line analysis revealed a good correlation (r = 0.722) between the mean test scores for the dominant hand at T0 and T1.

In our patients, the results obtained from the regression line analysis of the correlation between the mean 9HPT at T0 and T1 for the non-dominant hand showed a positive correlation, r = 0.588, compared to the mean 9HPT at T0 and T1 for the dominant hand where a correlation of r = 0.722 was obtained. The difference between dominant and non-dominant limbs reflects the correlation strength of the tests applied in dynamics, more precisely at the time of inclusion in the study and 12 months apart. There are also inverse situations, such as the one evidenced in the study by Wang et al., where an analysis was performed on a group of 4319 healthy subjects who were administered the 9HPT test. The results showed that the mean test execution time for the dominant hand was 22.5 versus 24.2 for the non-dominant hand [27].

These 9HPT performance results depend on several factors, including age, gender, brain dominance (right or left-handed), and the manual dexterity ability of one of the limbs. However, the most important factor influencing the quality of the 9HPT results is the presence of a motor deficit in one of the limbs.

To evaluate the cognitive impairment, the SDMT test was used, a highly prevalent psychometric tool used worldwide to assess the level of cognition in individuals with MS. One key benefit of this test is its ability to provide precise data on the speed of information processing. Sandry et al. conducted a study on a group of 661 patients with MS to evaluate different aspects of cognitive function using the SDMT test. They found that the SDMT test is highly effective in identifying cognitive dysfunction in MS patients and that a KMO analysis yielded a result of 0.86, supporting the validity of the study’s hypothesis [25].

The evaluation of cognitive performance in dynamics revealed that applying the regression between the SDMT score at T0 and T1 yielded a positive correlation coefficient of r = 0.753. This approach demonstrates that the proposed model for validation is both sensitive and specific in detecting cognitive degenerative alterations in patients with MS.

A recently published study analyzing the data from 14 clinical trial registries indicates that the SDMT is superior to the paced auditory serial addition test (PASAT) [26]. Therefore, using the SDMT test is a very accurate and precise approach that may be employed in both regular clinical practice and for monitoring the progression of the disease or defining the form of SPMS.

Considering the fact that the incidence of demyelinating diseases is constantly increasing due to the survival rate in this category of patients constantly increasing due to current diagnostic and treatment methods, the need to develop algorithms to monitor disease progression closely may represent a standard in the clinical practice of neurologists. Thus, in a study by Pham and coworkers, a mobile application of the SDMT test was developed with the role of self-assessment of cognitive function at home for MS patients. The mobile application was validated on 154 MS subjects and 39 healthy volunteers. The results of this study revealed a number of positive correlations between the SDMT score obtained using the mobile application and other tests measuring cognitive function and the measurement of brain lesions revealed using MRI.

In our study, the model created by means of the multivariate regression function obtained an R = 0.826 and *p* < 0.001. These values emphasize that the algorithm proposed for validation is methodologically specific and sensitive, showing a high degree of correlational power between the studied variables. Moreover, the algorithm demonstrates accuracy in terms of establishing the diagnosis of the SPMS phase.

An optimal diagnostic algorithm is one that can accurately identify both early and advanced forms of the disease

Pike et al. highlighted that the group of researchers classified patients into an “active/inactive” form, which served as the foundation for defining the SPMS form. They also identified age, disease activity, and EDSS score value as statistically significant factors that could potentially serve as markers of disease progression [28]. Our study found that multivariate linear regression analysis was effective in detecting progression to the SPMS form, specifically for tests that measured the level of motor impairment.

The SPMS form is strongly correlated with the greatest burden of illness impact. When compared to other forms of MS, an increase in the EDSS score over a period of three years was seen. These patients are more likely to progress to the SPMS phase, although there is currently no established method to describe this process due to the absence of sensitive and specific biomarkers that can distinguish between the RRMS and SPMS forms in the early stages of the disease [29].

The comparative analysis done in our study evaluated the validation of criteria proposed by a working group of the Romanian Society of Neurology and criteria proposed by expert groups from Spain, Karolinska, and Croatia for diagnosing SPMS. We chose these experts because they belong to experienced and well-known centers for studying MS and the population in those countries is similar to the Romanian one. The ROC analysis demonstrated that the criteria proposed by a working group of the Romanian Society of Neurology accurately assessed the degree of motor disability and effectively diagnosed SPMS, as they showed both sensitivity and specificity. However, the ROC analysis conducted to evaluate cognitive impairment using the SDMT test did not reveal significant levels of sensitivity and specificity (AUC: 0.616).

The ROC curve results regarding the application of the criteria proposed by the group of experts from Spain in relation to the analysis of the 9HPT, 25FWT, EDSS, and SDMT tests showed that the specificity and sensitivity are high for all the studied variables. This illustrates that the application of these types of tests is a sensitive method for detecting disease progression and could be part of a clinical algorithm that can be performed at least once yearly on a patient.

The ROC curve analysis comparing the criteria proposed by the Karolinska expert group with those proposed by the Romanian Society of Neurology revealed that only when using the EDSS score, an AUC value of 0.626, *p* = 0.024, was obtained. In this particular case, the sensitivity was found to be slightly to moderately significant. The decision algorithm for categorizing patients into a progressive form of disease according to the Karolinska criteria includes only information on age and EDSS score values, compared to the criteria proposed by the working group of the Romanian Society of Neurology, which include information on cognitive dysfunction, upper and lower limb motor deficit, and the measurement of global motor deficit dysfunction, quantified via the EDSS test.

The ROC curve analysis comparing the criteria proposed by the Croatian group of experts with those proposed by the Romanian Society of Neurology working group revealed that only the EDSS test demonstrated both specificity and sensitivity in accurately defining the form of SPMS. The AUC for the EDSS test was 0.950, indicating high accuracy, and the *p*-value was 0.001, indicating statistical significance. In contrast to prior consensuses, the criteria proposed by the Romanian Society of Neurology working group evaluate both motor and cognitive skills. The algorithm proposed for validation indirectly explains the pathophysiologic theories underlying the initiation of neurodegenerative processes and chronic inactive inflammation, which are specific elements of the SPMS phase. Statistically significant results were obtained for both components.

One of the limitations of this study could be that we focused exclusively on the clinical assessment and the EDSS, 9HPT, 25FWT, and SDMT assessment and did not include the imaging characteristics of demyelinating lesions and their disposition. Another limitation of our study is that we did not include data on age and disease duration in the decision process for classifying the type of SPMS, but categorizing a patient into the SPMS form considering such criteria may be falsely positive, as some patients may develop only a “pseudoprogression” of the disease.

The main limitations of this research to validate some clinical criteria are the following: the study group consisted of a relatively small cohort and a short follow-up period; thus, larger cohort studies and the inclusion of other clinical or paraclinical parameters such as serum biomarkers might be needed.

The future studies that could emerge from this research, although it is a relatively medium cohort of patients with a short follow-up period and with testing in the dynamics of some clinical parameters, should focus on larger cohorts and the inclusion of other clinical and paraclinical parameters, such as serum or MRI biomarkers or quality of life scales. Given the new disease-modifying therapies, this study opens the way for extensive research into progressive MS, a highly relevant topic in the next few years.

## 5. Conclusions

This study aimed to demonstrate the sensitivity and specificity of certain variables in diagnosing SPMS. Statistical analyses confirmed that the clinical algorithm proposed by the Romanian Society of Neurology working group is valid and can significantly aid in identifying patients at high risk of disease progression or those already in the SPMS stage.

Some of the statistical methods used in our study (the Kaiser–Meyer–Olkin, Bartlett’s test, the correlation matrix, and the Spearman correlation) revealed a statistically valid correlation between the tests used over a period of one year and indicated that they can serve as clinical markers for MS progression.

The initial comorbidity analysis showed that each test was statistically significant in relation to other clinical parameters. Spearman’s correlation demonstrated moderate to strong statistical correlations for all diagnostic “events” in the algorithm proposed by the Romanian Society of Neurology working group, and multivariate linear regression yielded significant results specifically for the EDSS, 9HPT, and 25FWT tests.

This study is the first in our region in Romania to analyze and validate key factors that can accurately define the diagnosis of SPMS. It offers valuable insights for improving early detection and targeted interventions in patients at risk of progression, and also it holds a practical significance, because these results might assist healthcare providers in better managing SPMS. Clarifying the real-world applications and implications for patient outcomes would enhance its significance. These findings lay the groundwork for more refined diagnostic tools and better management of MS in clinical practice.

## Figures and Tables

**Table 1:** Classification of clinical criteria defining the form of SPMS.

Criteria from Expert Groups in Spain	Criteria from Expert Groups in Karolinska	Criteria from Expert Groups in Croatia	Criteria Proposed by the Working Group of the Romanian Society of Neurology
- worsening of 2 points in the functional system; - <10 years of disease; - >20% time increase in 25FWT; - >20% time increase in 9HPT; - repeated falls; - worsening by at least 20% in the SDMT test.	- changes of EDSS scores of <4 or >4 in relation to age and EDSS score was the second criterion of the decision tree.	- progressive accumulation of disability after initial relapse of remittent disease; - an EDSS progression of 1 point for EDSS score up to 5.5 and 0.5 points for EDSS score ≥ 5.5, without relapse; - a pyramidal functional system score ≥ 2; - the absence of pregnancy.	- EDSS 1 for an initial EDSS score < 5 and 0.5 starting from an initial EDSS score of 5.5; - a minimum increase of 20% in completion time for either 25FWT or 9HPT; - SDMT (additional score) and a decrease of 3 points between the 2 assessments was considered pathological.

Abbreviation: expanded disability status scale (EDSS), timed 25-foot walk (25FWT), nine-hole peg test (9HPT), symbol digit modalities test (SDMT).

**Table 2:** Characteristics regarding the used tests in the studied population (results are in seconds).

Studied Parameter	Women	Men
EDSS T0	3.50 (2.00; 5.00)	3.82 ± 1.37
ESDD T1	4.00 (2.25; 6.00)	4.32 ± 1.64
9HPT mean dominant hand T0	28.26 (23.42; 32.35)	31.63 ± 10.68
9HPT mean non-dominant hand T0	28.80 (24.36; 34.88)	32.03 (23.10; 39.00)
9HPT mean dominant hand T1	27.71 (22.80; 31.00)	31.00 (23.98; 37.00)
9HPT mean non-dominant hand T1	30.00 (22.50; 34.96)	32.40 (23.75; 37.41)
25FWT mean T0	6.33 (5.12; 8.84)	6.50 (4.50; 9.42)
25FWT mean T1	6.25 (4.64; 10.45)	7.38 (4.85; 10.29)
SDMT T0	30.88 ± 13.38	30.60 ± 13.25
SDMT T1	23.00 (15.00; 35.00)	24.54 ± 10.79

**Table 3:** Results of Bartlett test.

	KMO and Bartlett’s Test	
Kaiser–Meyer–Olkin		0.683
	”Approx. Chi-Square” Index	1022.059
Bartlett’s Test of Sphericity	Degree of statistical difference-df	45
	Level of statistical significance-Sig.	<0.001

**Table 4:** Results in the correlation matrix test.

Correlation	EDSS T0	EDSS T1	9HPT Mean T0-D	9HPT Mean T0-ND	9HPT Mean T1-D	9HPT Mean T1-ND	25FWT Mean T0	25FWT Mean T1	SDMT T0	SDMT T1
EDSS T0	1.000	0.915	0.327	0.402	0.380	0.344	0.172	0.291	−0.371	−0.379
EDSS T1	0.915	1.000	0.329	0.387	0.389	0.294	0.239	−0.344	−0.379	−0.427
9HPT mean T0-D	0.327	0.329	1.000	0.644	0.894	0.665	0.160	0.178	−0.349	−0.360
9HPT mean T0-ND	0.402	0.387	0.644	1.000	0.565	0.767	0.171	0.159	−0.404	−0.389
9HPT mean T1-D	0.380	0.389	0.849	0.565	1.000	0.553	0.146	0.185	−0.259	−0.344
9HPT mean T1-ND	0.344	0.294	0.665	0.767	0.553	1.000	0.109	0.101	−0.331	−0.345
25FWT mean T0	0.172	0.239	0.160	0.171	0.146	0.109	1.000	0.920	−0.235	−0.228
25FWT mean T1	0.291	0.344	0.178	0.159	0.185	0.101	0.920	1.000	−0.224	−0.227
SDMT T0	−0.371	−0.379	−0.349	−0.404	−0.259	−0.331	−0.235	−0.224	1.000	0.868
SDMT T1	−0.379	−0.427	−0.360	−0.389	−0.344	−0.345	−0.228	−0.227	0.0868	1.000

Legend: HPT mean T0-D = 9HPT mean T0 = 0.644/T1 = 0.665 dominant hand; 9HPT mean T0 = 0.644/T1 = 0.767; ND = non-dominant hand.

**Table 5:** Covariance of the studied parameters.

	Covariance			
	Factor Matrix		Rotated	
	Initial	Extracted	Initial	Extracted
EDSS T0	2.244	0.575	1.000	0.256
EDSS T1	3.069	0.878	1.000	0.286
9HPT mean T0 dominant hand	109.042	89.407	1.000	0.820
9HPT mean T0 non-dominant hand	108.900	78.904	1.000	0.725
9HPT mean T1 dominant hand	134.773	101.338	1.000	0.752
9HPT mean T1 non-dominant hand	160.154	127.305	1.000	0.795
25FWT mean T0	37.393	32.079	1.000	0.858
25FWT mean T1	77.196	72.204	1.000	0.935
SDMT T0	176.231	166.203	1.000	0.943
SDMT T1	163.228	150.331	1.0	0.921

**Table 6:** Spearman correlation results.

Spearman’s Rho Coefficient		Proposed Criteria for the Detection of the SPMS Form
EDSS ”event”	Correlation coefficient	0.364
	Sig. (2-tailed)	<0.001
	N	120
9HPT ”event”	Correlation coefficient	0.525
	Sig. (2-tailed) N	<0.001 120
25FWT ”event”	Correlation coefficient	0.574
	Sig. (2-tailed)	<0.001
	N	120
SDMT ”event”	Correlation coefficient	0.231
	Sig. (2-tailed) N	0.011 120

**Table 7:** Beta coefficient in relation to the “event” 9HPT, 25FWT, SDMT, EDSS.

Studied Variables	Non-Standardized Coefficient	Standardized Coefficient		Sig.	95.0% Confidence Interval B			Collinearity Tests
	B	Std.	Beta		Lower	Upper	Tolerance Inflation Factor	
		Error			Limit	Limit		
(Constant)	0.093	0.049		0.061	−0.005	0.191		
EDSS	0.381	0.070	0.295	<0.001	0.242	0.519	0.951	1.052
9HPT	0.581	0.065	0.492	<0.001	0.453	0.709	0.933	1.072
25FWT	0.554	0.060	0.504	<0.001	0.435	0.672	0.946	1.058
SDMT	0.028	0.056	0.028	0.625	−0.084	0.139	0.880	1.137

## Data Availability

The data presented in this study are available on request from the corresponding author due to the privacy of the data.

## Supplementary Materials

The following supporting information can be downloaded at: https://www.mdpi.com/article/10.3390/brainsci14111141/s1, Figure S1: Correlation between the Scree scales; Table S1: ROC curve results on the application of the criteria proposed by a working group of the Romanian Society of Neurology; Table S2: Results of the ROC curve when applying the criteria proposed by the Spanish expert group; Table S3: Results of the ROC curve when applying the criteria proposed by the Karolinska expert group; Table S4: Results of the ROC curve on the application of the criteria proposed by the Croatian expert group.
