# Supplementary material for: Validation of a Set of Clinical Criteria for the Diagnosis of Secondary Progressive Multiple Sclerosis
Source: Brain Sci. 2024 Nov 14;14(11):1141. doi: 10.3390/brainsci14111141 (PMC11591908; doi:10.3390/brainsci14111141)
Supplement: Supplementary file 1 [file brainsci-14-01141-s001.zip › brainsci-3288225-supplementary.pdf]

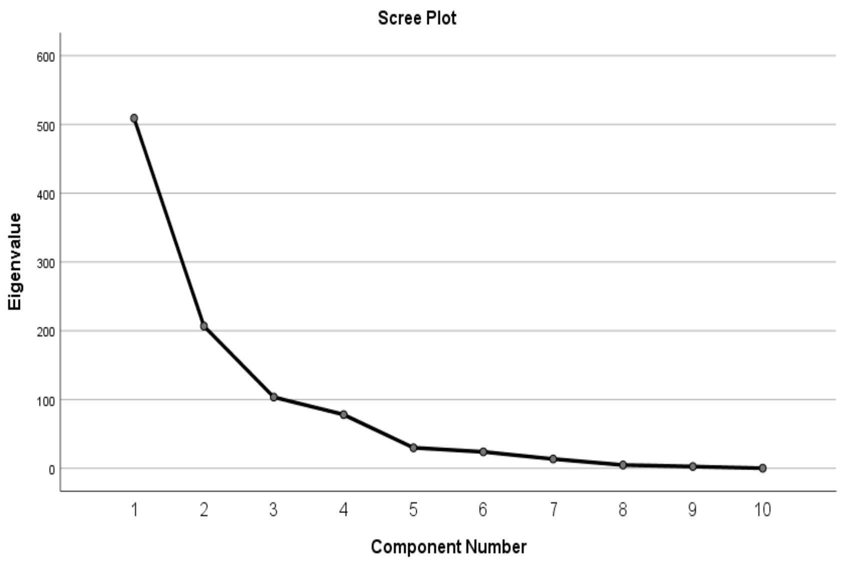

Figure S1. Correlation between the Scree scales

Table S1. ROC curve results on the application of the criteria proposed by a working group of the Romanian Society of Neurology

Area under curve					
Studied variables	Area	Std. Error[a]	Asymptotic Sig.[b]	Asymptomatic 95% confidence interval	
				Lower limit	Upper limit
EDSS ”event”	.641	.050	.008	.543	.740
9HPT ”event”	.722	.047	.000	.631	.814
25FWT ”event”	.761	.044	.000	.674	.848
SDMT ”event”	.616	.052	.029	.515	.717

Table S2. Results of the ROC curve when applying the criteria proposed by the Spanish expert group

Area under curve					
Studied variables	Area	Std. Error[a]	Asymptotic Sig.[b]	Asymptomatic 95% confidence interval	
				Lower limit	Upper limit
SDMT ”event”	.744	.092	.151	.564	.923
25FWT ”event”	.863	.053	.032	.759	.967
9HPT ”event”	.893	.043	.020	.808	.978
EDSS ”event”	.910	.038	.015	.837	.984

Table S3. Results of the ROC curve when applying the criteria proposed by the Karolinska expert group

Area under curve					
Studied variables	Area	Std. Error[a]	Asymptotic Sig.[b]	Asymptomatic 95% confidence interval	
				Lower limit	Upper limit
EDSS ″event″	.626	.056	.024	.516	.736
9HPT ″event″	.471	.055	.603	.363	.579
25FWT ″event″	.538	.056	.498	.428	.648
SDMT ″event″	.490	.056	.862	.381	.600

Table S4. Results of the ROC curve on the application of the criteria proposed by the Croatian expert group

Area under curve					
Studied variables	Area	Std. Error[a]	Asymptotic Sig.[b]	Asymptomatic 95% confidence interval	
				Lower limit	Upper limit
EDSS ″event″	.950	.031	.000	.889	1.000
9HPT ″event″	.510	.071	.888	.370	.650
25FWT ″event″	.595	.072	.181	.453	.737
SDMT ″event″	.485	.071	.833	.346	.624
